# Introduction: Public Health Genomics—Anthropological Interventions in the Quest for Molecular Medicine

**DOI:** 10.1111/maq.12055

**Published:** 2013-11-08

**Authors:** Karen-Sue Taussig, Sahra Elizabeth Gibbon

**Affiliations:** Department of Anthropology, University of Minnesota; Department of Anthropology, University CollegeLondon

**Keywords:** genomics, public health, ethnography, race, environment

## Abstract

We introduce this special issue of Medial Anthropology Quarterly on public health genomics by exploring both the unique contribution of ethnographic sensibility that medical anthropologists bring to the study of genomics and some of the key insights offered by the essays in this collection. As anthropologists, we are concerned with the power dynamics and larger cultural commitments embedded in practices associated with public health. We seek to understand, first, the broad significance of genomics as a cultural object and, second, the social action set into motion as researchers seek to translate genomic knowledge and technology into public health benefits.

For too long, discussions concerning the ethical, legal, and social implications of genomics have abstracted questions of power and the production of knowledge out of their frames of reference to focus on individual and individualizing issues such as autonomy, choice, consent, and “cultural competence.” Though often productive, such analyses are severely limited, most fundamentally by an implicit understanding of the social as something that is somehow subsequent to science.

These discussions have developed over the past two decades, decades that have been marked by an explosion of knowledge in the life sciences, particularly knowledge related to the mapping of the human genome and subsequent efforts to realize the goal of using this new knowledge to improve human health. In these efforts, we see the emergence of a complex social terrain in formation, characterized by the rethinking of earlier scientific orthodoxies about biology that, in turn, have opened up an imaginative space for envisioning and developing new medicines and biotechnologies that might animate bodies and extend life in new ways.

In this space, we find genomics shifting between a focus on lab-based mapping and the uptake of early findings in such practices as prenatal testing (see, e.g., Inhorn 2008; S. Kahn 2000; Rapp 1999; Rothman 1993; Taussig 2009) to the hype and hope-filled promise of personal medicine (see, e.g., Borup et al. 2006; Brown 2003; Fortun 2008; Hedgecoe 2004; Rajan 2005; Taussig 2005, 2007) and to an emerging focus on public health and the complex interactions among epigenetics, epidemiology, infectious disease, environmental exposures, and human biological variation (Landecker 2007; Lock 2005; Shostak 2005; Taussig 2005, 2007). These interlinkages are no longer confined to or arise solely in the context of developed countries but are unfolding in global and transnational arenas with often uneven, inequitable, and unexpected consequences (Bharadwaj and Glasner 2009; Gibbon and Reynolds 2009; Gottweiss et al. 2009; Rajan 2005).

## Medical Anthropology and Public Health Genomics

The relationship between genetics and public health is, of course, not in itself novel, but it has a troubling history in its previous union in eugenics movements (Kevles 1995 [1985]; Lombardo 2010; Proctor 1988, 1995). In reaction to the eugenic excesses of the mid-20th century—from the Holocaust to widespread mandatory sterilization programs at least in the United States and Scandinavia—after World War II, life scientists focusing in this area worked hard to disinvest the science of genetics with such connotations and establish it as a discipline focused on basic science and on disease problems with a goal of medical application (Keller 1992).

Indeed, the rationale for the more than $3 billion funding for the Human Genome Project, as well as subsequent public and private support for genomics research, has been that such knowledge would pay off in the form of valuable interventions into human health, frequently couched in terms of an ultimate goal of medicine tailored to individual risks and susceptibilities. Nevertheless, a tension persists between a widespread commitment to this view of contemporary genomics (see, e.g., Schaefer et al. 2009) and recognition that eugenic thinking has had a continuous place in science and medicine right up to the present (Duster 1990; Kerr 2003; Reardon 2005; Taussig et al. 2003).

The cultural anxieties that have accompanied the establishment of genetics as big science stem, in part, from the on-going eugenic possibilities that emerge with such knowledge. With other anthropologists (e.g., Fortun 2005; Hoeyer 2008; Petryna et al. 2006; Rapp 1999), we contend that responses to such anxieties are too often framed in the language of a traditional bioethics.

Although we recognize that bioethics is a diverse international field with a range of approaches, which in some instances aims to incorporate broader sociopolitical dimensions (see, e.g., Parker and Bull 2009), we find that what we are describing as a “traditional” approach seems most frequently foregrounded in bioethics committees and in policy. This bioethics tends to focus on concerns for individual/consumer choice by emphasizing the importance of informed consent with respect to clinical trial research or the application of novel genetic knowledge or technologies. It is a particular framing of ethical problems that implicitly reduces fears about contemporary genomics to discrete concerns with individual autonomy and choice within specific research agendas and clinical practices, rather than on the multiple specific dimensions, interactions, and processes through which such phenomena are articulated and pursued. Such a focus tends to obscure the power dynamics and the larger cultural commitments embedded in this domain of contemporary social life.

As anthropologists, we are concerned precisely with these cultural commitments and the practices that simultaneously produce and reinforce them. In particular, we seek to understand, first, the broad significance of genomics as a cultural object and, second, the social action set into motion as researchers seek to translate genomic knowledge and technology into public health benefits.

A highly interdisciplinary group of scholars in a wide array of national contexts is working to understand this emerging “naturalcultural” terrain, defined by the simultaneous production of the biological and the social (Haraway 2003; Helmreich 2009; Marks 2009), in which the growing and uneven scope of genetic technologies and knowledges is informing and interacting with different institutional, national, and transnational arenas of health care. Here we find contemporary concerns with genomics and public health now emerging through the lens of epigenetics and a contemporary concern with gene–environment interactions. The complex and changing nature of genetic knowledge in relation to seemingly older fields of inquiry such as epidemiology, which now link to and are reframed by what appear to be novel areas of investigation such as epigenetics or toxico-genomics, raises new challenges for social science and cross-disciplinary investigation (see Fortun and Fortun 2005; Shostak 2005).

The essays in this collection illustrate that medical anthropologists bring at least three important and intersecting commitments to investigating these problems.

First, they have an interdisciplinary orientation that insists that *all* knowledge—including biomedical knowledge and the scientific knowledge from which it draws—is social knowledge that simultaneously requires and demands a deep understanding of both the science and the medicine involved in a particular area of study (Young 1982; see also Lock et al. 2000; Martin 1987, 1994; Rapp and Disotell 2003).

Second, like other anthropologists, medical anthropologists employ what Daniel Segal and Sylvia Yanagisako (2005) named a “complex cluster of intellectual dispositions” in orienting their investigations. Segal and Yanagisako trace these dispositions back to Boas's critique of race and racial science.^1^ They include a “propensity to be suspicious in the face of the convergence of scientific claims, on the one hand, and both social prejudice and ethnocentrism, on the other” as well as (echoing Young) “a critical empiricism” that seeks to “grapple with, rather than to deny, the difficulties raised by the recognition that human observation is always already socialized” (Segal and Yanagisako 2005:13). They also make an important point about the value of comparison, arguing that another Boasian disposition anthropologists bring to their analyses is an “epistemological stance” that recognizes “there are no insignificant human ‘cases,’ meaning that knowledge of ‘humanity’ must be based as much on the exotic as on the familiar” (Segal and Yanagisako 2005:13). Such dispositions are crucial for understanding genomics as a cultural object in general and, regarding the focus of this collection in particular, for understanding how social action is set into motion in efforts to build the knowledge and practices of a public health genomics. Anthropologists achieve this by highlighting the cultural commitments that underlie these efforts and by providing crucial comparative perspectives on the changing nature of genomics as it is emerging.

Third, medical anthropologists work with a commitment to fine-grained ethnography and to developing a deep understanding of the world views of those with whom we work. As Susan Reynolds Whyte points out, ethnography is “the test” (2009:10) that enables the illumination of the everyday experiences of diverse social actors. In the essays included in this collection, we see these actors working to realize and/or benefit from molecular medical knowledge and the production and application of public health genetics.

To the extent that anthropological work reflects these three commitments, it contributes unique insights to the effort to understand this complex social field. In so doing, the articles in this issue illustrate a pathway past current public health, bioethical, and social science preoccupation with adding social context as the solution to understanding the dynamics or social impact of emerging genomic knowledge. Instead, they point to the multiple specific dimensions, interactions, and processes through which the social is never after the fact of technological innovation. Indeed, we contend that simply adding social context as after the fact offers too simplistic renderings of context and of culture.

As the contributions to this special issue illustrate, efforts to develop public health genetics also open up new questions for medical anthropology about nature and culture, citizenship, inequities in access to and provision of health care, institutional ethics, and transnational flows of capital. These phenomena, in turn, offer new conceptual and methodological challenges for the discipline about how to engage with, include, and address questions of biology, of nature, and of the scientific in an evolving era of genomics.

Our interest in these issues provided the impetus that has resulted in this collection. The essays were initially developed for a session we organized on public health genomics for the 2009 meetings of the Society for Medical Anthropology for which Susan Lindee and Rayna Rapp provided commentary.^2^ In addition to what is included here, papers were presented by Karen-Sue Taussig, Margaret Sleeboom-Faulkner, and Michael Montoya. Peter Fry also developed a paper but was unable to travel for the conference. Although these are not included here, we discuss some of the significant points made in the presentations, as well as other relevant research, to illuminate the broad themes and cross-cultural perspective anthropological work in this area addresses.

### The Case of Breast Cancer and BRCA Genomics

There are numerous illustrations and examples where the demand for interdisciplinary engagement and communication becomes evident and urgent. One such example is provided by the rapidly expanding and dynamic field of BRCA genomics, which offers a pertinent reminder of the necessity and challenge of cross-disciplinary work. The discovery of the two so-called breast cancer genes in the mid-1990s and the ensuing institutionalization of BRCA knowledge-practices and accompanying medical techniques for assessing genetic risk, including predictive genetic testing, has developed rapidly in Euro-American societies; a trajectory of hype and hope that has paralleled (and intersected with) the public discourse surrounding the human genome project in the late 1990s.

The rapid expansion of the field of BRCA medicine has, not surprisingly, been an area of interest for social scientists, with studies examining patients’ perceptions of risk and questions of identity for those caught up with this novel field of health care (Finkler 2000; Gibbon 2007; Gibbon et al. 2010; Gibbon et al. In press[Bibr b24]; Hallowell 1999). Others have turned attention to the institutional cultures and practices that surround the application of this novel medical intervention, often linked to the specificities of public health in different cultural contexts (Bourret 2005; Löwy and Gaudillière 2009; Parthasarathy 2007) but also often associated with cultures of activism (Gibbon 2008) or religious and community organization (Kampriani 2009). A growing body of work is also exploring the meaning of BRCA medicine for specific populations, including underserved groups in the United States and Ashkenazi Jewish women (Mozersky 2012; Mozersky and Galen 2010).

It also has become increasingly obvious that the application of BRCA testing in clinical arenas is taking place in a terrain of on-going medical and scientific debate and discussion that is only just beginning to understand the complexity of disease now related to genetic, epigenetic, and gene–environment pathways. The recognition of this complexity makes research and medicine linked to the BRCA genes a highly mobile arena of scientific inquiry that poses continuous challenges for medical application *and* social scientists interested in understanding these research trajectories, their clinical dynamics, and the consequences of ongoing medical uncertainty for patient identity and health practice.

New avenues of medical and scientific research demand attentive and engaged social science inquiry. This includes the development of treatment interventions for BRCA carriers and the use of knowledge about the BRCA genes to develop treatment pathways for sporadic cancers (Bourret et al. In press[Bibr b4]) as well as the growing interest in “founder mutations” connected to the BRCA genes in different national or geographical arenas.

These latter research initiatives are directly associated with a discourse on public health linked to the hope of developing targeted treatment intervention or cheap testing technology that can be made more widely available to underserved populations and communities. In fact, in the broader post-genomic space of breast cancer now emerging, the social and scientific complexity of the dynamics between BRCA, breast cancer, and public health genomics is becoming apparent. This is particularly so now that ancestry, race/ethnicity, and inequities and ethnic disparities in breast cancer incidence and mortality have begun to inform and (for some) provide a justification for scientific research (see, e.g., Fejerman et al. 2008).

These shifting research dynamics around the BRCA genes illustrate the vital importance and necessity of much closer collaboration between the natural and social sciences. As other commentators have also noted (e.g., Lock 2005; Lock and Nugyen 2010; Montoya 2011; Rapp and Disotell 2003), the life sciences constitute an arena that is producing both profound challenges and novel opportunities for medical anthropology that require us to think seriously and reflexively about the meaning of interdisciplinarity.

## What Is at Stake? An Overview

The articles in this special issue address these different yet intersecting issues at the heart of efforts to translate genomic knowledge into clinically useful interventions for public health through two primary themes: First, how is the interface between different aspects of genomics (e.g., epigenetics, epidemiology, human biological variation, predictive and prenatal/neonatal testing) and public health being configured in diverse national arenas? How do the state, institutional actors, and citizens become positioned in relation to emergent genomic practices aiming to develop molecular medicine? Second, what are the frameworks (theoretical and methodological) for analysis in medical anthropology when the biological and the social are being framed in relation to genomics at the same time that epigenetics and environmental factors are becoming more central to genetic science?

In addressing these issues, the authors focus on a wide variety of emerging aspects of genomic medicine and public health interventions as these relate to diverse disease conditions across a broad range of international/transnational cultural arenas. These areas include Cuba, the United States, the Caribbean, and Europe.

The changing space of genomic/postgenomic knowledge and technology as it relates to common complex conditions such as asthma and breast cancer provides one cross-cutting theme for the collection. Of particular interest to several of the authors are the ways that new concerns about environmental factors are being articulated, performed, and mobilized in relation to the (often) contradictory demands and concerns of genomics as public health.

The politics of “race” provides a second topical theme that connects the interests of a number of contributors. Interest centers on how race plays out as part of specific seemingly older *and*, simultaneously, more novel public health interventions in very different national arenas, such as Barbados and the United States, where the historical and social meaning of race/ethnicity/identity have been very differently constituted.

The role and meaning of “publics” or “community” and “citizenship” is a third theme that is taken up and addressed by a number of the authors. Contributions focus on the way these often intersecting ideas/concepts and practices become invoked, enlisted, and coproduced through genomic interventions as “personalized medicine” unfolds in diverse arenas and as efforts harmonize the global governance of genomics, often through ethical norms, have diverse effects on local practice.

## Environment, Epidemiology, and Community

Since the inception of public health as a modern project, the environment, environmental factors, and the control of these have been central concerns for the discipline. From sanitation projects that work to prevent the proliferation of microbial life to vaccination campaigns that moderate the body's encounter with hitherto deadly viruses, public health has sought to ensure human health in relation to the environment. Such efforts frequently occur as national projects focused on a particular nation-state, as a component of colonialism, and/or as an aspect of development (see, e.g., Latour 1983, 1988; Nash 2007; Quirke and Gaudillère 2008; Rosenberg 1976).

Environmental health scientists have diverse and often extremely broad views about the environment, including everything from a body's exposure to a nearly infinite range of organic and synthetic compounds to more or less complex renderings of socioeconomic factors such as diet, exercise, education, stress (including that associated with poverty and discrimination), and so on (Fortun and Fortun 2005; Frickel 2004). Conceptualization of the environment can become even more complicated in the context of public health genomics. For public health geneticists seeking to understand the relationships among genes and environments, the environment might also mean the environment inside the nucleus of a cell, the cell itself, a fetus, a uterus, radiation exposures, and so on.

The essays in this collection take up the question of environment in diverse ways. In the postgenomic era, Ian Whitmarsh suggests, conceptualizations of the environment are central to new configurations of genetic expertise, public health, and governance. For Whitmarsh, who focuses on asthma in Barbados, a country with one of the highest levels of asthma in the world, what precisely constitutes the environment is a site of contestation in which community health, scientific expertise, and the dangers of modernization are at stake. Whitmarsh illustrates that, in Barbados, public health practitioners concerned with asthma increasingly are coming to rely on genotyping technologies as a means of interpreting an environment comprised of household allergens that interact with genes associated with allergic response. This approach challenges community understandings of asthma as a result of modernity, related to an environment characterized by road work, dietary changes, insecticides, and vehicle exhaust. In so doing, genomic research turns the meaning of environment from causative to background, from demographic to individualized.

Taking up the question of communities in his presentation at the Society for Medical Anthropology meeting, Michael Montoya reminded us that for decades social and behavioral approaches to health research have been concerned about the interconnections between environments and behavior. Contemporary efforts to translate genomic and other biological information into clinically useful interventions now are turning to community-based research practices. In examining this phenomenon, Montoya argues that community knowledge is necessary to understand both illness and basic biological processes. At the same time, he suggests that it remains to be seen whether the problems in the environment, and those experienced by the humans within it, will direct knowledge production activities or will merely be used as data points, blood samples, or trial specimens.

Epidemiology is a core component of contemporary public health practices. In her essay focusing on genetic epidemiology, Susanne Bauer suggests that with genetics, scientists hoped to “open epidemiology's ‘black box between [environmental] exposure and disease.’” Echoing one of Whitmarsh's points, Bauer illustrates that in this modeling, what precisely “environment” means is being transformed. Bauer argues that in the context of genetic epidemiology the environment has come to have functional significance. To grasp its effects, ever larger epidemiological studies are required, creating a demand for ever larger datasets, thereby changing the ways epidemiology is practiced.

It is precisely this concern to have access to large datasets incorporating both genetic and environmental information that have set into motion diverse efforts to develop biobanks, a topic to which we will return below. Here, we note that they are a key site for efforts aimed at understanding the relationships among genes and environments that are at the heart of contemporary public health genomics. In working to develop this understanding, questions of population, biological variation, and race also become key themes.

Biobanking does not yet figure explicitly under the remit of community genetics initiatives described by Sahra Gibbon in her examination of Cuban interventions in public health genomics. However, the local organization of genetic services that is targeted at the level of the family forms the basis on which the explicit aim of aligning social and biological factors may be used to address a range of growing health problems such as breast cancer. A concern and interest with social factors points to a tension that speaks to the broader geopolitical dynamics in which Cuba is situated.

On the one hand, we see a desire to situate community genetics within a modernist narrative, where growing investment in an expanding and competitive arena of biotechnology and science can address cutting-edge paradigms of genomic research that, at the very least, aim to include the role of the environment as part of a commitment to addressing public health problems. On the other hand, day-to-day work in community genetics that aims to incorporate health concerns and initiatives into a wider understanding of the social context of people's collective lives acts as an implicit critique of individualized approaches to health care in ways that question the moral mantra of genomics as personal medicine.

## Population, Biological Variation, and Race

Virtually by definition, public health has focused on questions of populations with regard to health, making various forms of population stratification one of its primary practices. Although it has long been recognized that many of the health disparities of concern to public health practitioners fall along racial lines, diverse explanations for such differences have been offered. There is a rich history in medicine of ideas about diseased black bodies (see, e.g., Hammonds 2002; Reverby 2009; Wailoo 2000; Washington 2006) or of ethnically identified ghettos as spaces of contagion (Craddock 2004).

Another narrative about racially stratified health disparities focuses on the social, suggesting that social conditions, including poverty, education, and racism, underlie such phenomena. Yet, as genomics has emerged as a paradigm through which to understand human health, concerns about these social factors often appear to fade as researchers focus on genetic differences that may contribute to a particular disease. Troy Duster, for example, points to the phenomenon of the high incidence of hypertension among African Americans (though not in Africans living in Africa).

A classic 1991 study of hypertension published in the *Journal of the American Medical Association* argued that stress, not anything biological or genetic, was the major factor contributing to the increased risk of hypertension for this population (Klag et al. 1991 cited in Duster 2007). But, as Jonathan Kahn (2005) has illustrated, at least by 2002 researchers were beginning to argue that heart failure, for which hypertension is a major precursor, was a different disease in African Americans rooted in underlying biological or genetic differences (see, e.g., Yancy 2002).^3^

Ironically, while the 2000 announcement of the completion of a first draft of the human genome was suffused with the idea that racial differences cannot be discerned at the molecular level, numerous scholars have noted that in the postgenomic era we are seeing the powerful reemergence of biological conceptions of race in medicine (Braun 2002; Duster 2005; Fullwiley 2007b; Goodman 2000; J. Kahn 2003, 2005; Lee 2009). Kahn argues that we should be concerned about a shift in understandings about the stratification of health along racial lines from a focus on disparities to an emphasis on difference both because such a shift reinscribes race at the molecular level and because, in so doing, it might undermine efforts to address health inequities (Fullwiley 2007b; Kahn 2005).

Analyzing the cultural logics behind the 1995 emergence of a discourse on sickle cell anemia along with black activism in Brazil, Fry (2011) reminds us of the fluidity of racial categories across diverse contexts (see also Fullwiley 2007a; Tapper 1999; Trouillot 1991). Fry's chapter seeks to solve the puzzle of why sickle cell anemia continues to be associated with the black body although its transmission as a recessive trait in a classic Mendelian fashion has been understood since 1949. Fry argues that understanding sickle cell anemia as a “natural symbol” of a “black race,” as opposed to a “white race,” helps us understand the emotional and political commitment of Brazilian black activists to a racial/ethnic understanding of the condition despite Brazilian scientists’ arguments to the contrary. He suggests that more than anyone else, these activists believe that Brazil is a society of blacks and whites but that they have been unable to “convince enough browns and mulattos to join common cause in a mass political movement” (2011:169).

As Peter Fry illustrates, it is a situation that reveals the way a very particular sociohistorical context, where (in the influential logic of Gilberto Freyre^4^) nationhood tied to a Brazilian collective identity as *miscegenacão* or mixed race is being challenged on multiple fronts. That is, it is being challenged by black Brazilian activists who rely on sickle cell as a natural symbol in their quest to mobilize broader identification with a black racial category and, conversely, by high-profile genetic studies in Brazil that promote the nation as constituted by uniquely different individuals.

The contributions to this volume take up issues of population, human biological variation, and/or race. In her investigation of public health genomics, Susanne Bauer interrogates how, with epidemiological techniques, molecular data are connected to population categories such that data on genetic variation are added as “markers of susceptibility” to epidemiological modeling. Such modeling is intended to create personalized recommendations for health promotion based on individualized risk, but Bauer shows that ultimately such individualized assessments are based on group-specific risk estimates that, when taken to the clinic, lead to specific profiling practices.

Ancestry, admixture, and neoliberal consumer identities are central themes in Sandra Lee's analysis of the process of racialization in a new domain in contemporary genetic practice: companies such as Navigenics, 23andMe, and deCODEme, which offer direct-to-consumer genetic tests. Her essay is, in part, a story of inscribing race in genes through DNA ancestry practices that rely on population sampling understood through the idea of continental ancestry. In this frame, samples from a Yoruba population in Nigeria come to be understood as “African” in spite of the great genetic diversity on the continent, while samples from a Han Chinese population in Denver come to signify “Asian.”

Lee's essay offers a warning about just whom any clinical translation of genomic knowledge might benefit. She illustrates how the history of population sampling practices, including the reluctance of many in communities of color to participate in research for a variety of reasons (e.g., a history of abuse), shape the results individuals can obtain from the genetic tests these companies offer. She notes that when comparing all the tests and their relevance to European versus non-European populations, it appears that over 93% are applicable only to Europeans. Pointing to the implications of this phenomenon for public health, Lee argues that the evidence suggests that it is unlikely that the clinical translation of genetic data will have much impact on the health disparities that burden communities of color. Here we find a politics of inclusion/exclusion affecting diverse communities and questions of citizenship in efforts to develop public health genomics.

## Publics, Communities, and Citizenship

Concern about developing public health genomics occurs in a gap between the promises genome scientists have made since the inception of the Human Genome Project to create dramatic new interventions into human health and its failure, to date, to realize these promises (Collins 2010). The now rather extensive literature on biobanking indicates how efforts to close this gap depend on access to large datasets that link genetic material to family histories, medical records, and environmental exposures. This literature focuses our attention on the specifics of the politics of personalized medicine (Hedgecoe 2004; Hoeyer 2008; Hoeyer and Tutton 2005; Taussig 2005, 2007), pointing to emerging forms of citizenship that turn every person into a potential research subject (Taussig 2005, 2007) and even may obligate them to participate in research as a duty of citizenship (see, e.g., Schaefer et al. 2009).

It is clear that what has been described as a form of “biological citizenship” is not uniform but variably and unevenly taken up across diverse social contexts (Gibbon and Novas 2008; Gibbon et al. 2010) in ways that can involve the state in more or less explicit ways (Raman and Tutton 2009). Sleeboom-Faulkner (In press)[Bibr b83] describes a Chinese effort to develop biobanks, including one in Taizhou, in Jiangsu Province, which has been described as “the world's largest human genetic biobank.” By creating and controlling access to this valuable resource, Sleeboom-Faulkner argues, the Chinese state is able to establish China as a crucial site of scientific competition and collaboration.

A recent special edition of *Genetics and Society* edited by Herbert Gottweiss (2009) is focused on Asia and illustrates the importance of considering the complex ways that neoliberal subjectivities and state interventions in biopolitics are often necessary components of diverse and expanding areas of genomic medicine. Nevertheless, Sleeboom-Faulkner explores how rapidly expanding fields such as biobanking cannot be understood simply in terms of Western concepts of neoliberal citizenship. She suggests that biobanking in China takes place in a hybrid context that includes deeply rooted histories of scientific determinism *and* a global arena in which competition and collaboration have become the guiding idioms of the state's investment in genomics. In so doing, she argues that this complex set of dynamics animates initiatives such as the Taiizhou biobanking project where, despite inadequate health care, people are prepared to donate their DNA and tissue as collective members of a *modern* Chinese nation. As Susan Greenahalgh points out: “The story of biopolitics in the molecular age is more complex and collective than we had thought” (2009:205).

The neglected role of the state in configuring genetics as public health is addressed explicitly by a number of essays in this collection. Most obviously, this is explored by Sahra Gibbon in her article on Cuba, where the state has traditionally been overdetermined in analysis of a socialist society. For Gibbon, the lens of inquiry is oriented to on-the-ground experiences, practices, and perspectives of health professionals caught up in the emerging field of Cuban community genetics.

Exploring how deeply held commitments are articulated in public and personal narratives by or about health professionals, Gibbon illustrates how an ethical and emotionally infused orientation to collective health care *and* a reading of genetic interventions as a modern “gendered” vocation inform the day-to-day practices of community genetics and the interactions between health professionals and publics. She shows how it is these dynamics that explain the interest in, and ultimately the difficulties surrounding, fledgling attempts by community genetic practitioners to address complex adult onset diseases such as breast cancer. A procedure such as testing for the two BRCA genes, which in Euro-American societies has come to exemplify the success of genomics and fuel the hope of genomics as personal medicine, is, in the practice and discourse of Cuban community genetics, “out of place.” With some difficulty, it is transmuted by health professionals into interventions that more easily fit the paradigm of collective public health.

Importantly, this engagement with genomics as public health in what has been seen as a socialist society makes evident that what is required is a perspective in which the distinctions between top down or bottom up, neoliberal and socialist, or traditional/modern are replaced by a commitment to considering the role of the state as part of a lived social and cultural process. The zones of “cultural friction” (Tsing 2005) constituted by the global/local/national processes that inform public health genomics in settings such as Cuba and other Western contexts cannot easily be parsed into simplistic dualities (see also Ong 2006). They must, as the pieces in this collection demonstrate, themselves be examined as necessarily relationally produced through concrete social, political, and historical processes that are always subject to unexpected and unstable articulation and engagement.

Citizenship, however, takes on an entirely different meaning in the U.S. context. In her presentation, Taussig examined a range of efforts to engage various publics in what she calls genetic thinking and practice. These efforts include a project to engage every citizen in Vermont in a conversation about genetics and ethics in the 1990s, a project to educate Native American college and university students in genetics, and a contemporary project to conduct a national series of town hall meetings about developing a national biobank. The diversity of these efforts—in regard to their strategies, the people they seek to engage, and their rendering of subjects in relation to the state and to medical research—is striking. Taussig suggests that there has been a shift from an earlier period of bottom-up community participation to a top-down model of engagement initiated by the National Institutes of Health and an emerging conception of participation in medical research as an obligation of citizenship.

In examining these issues from different regional and topical perspectives, this special issue is an important and timely contribution to the emerging area of public health genomics, providing an important cross-section of research that can speak about and to the changing role and demands on medical anthropology concerned with the politics of public health. Seen through the lens of three key arenas of engagement that includes novel concerns with the environment, the politics of race as it plays out in different cultural/historical contexts, and the role of publics, communities, and citizenship, the essays illustrate the diversity of cultural and sociopolitical issues and problems raised by the conjunction of genomics and public health.

Such developments constitute a moment of opportunity and challenge for medical anthropology that demand both urgent engagement and critical reflection. Meeting these multiple demands will not be easy, but we contend that these articles and similar future efforts will go some way to constituting and sustaining the vitality and relevance of the discipline of medical anthropology in the 21st century.

## Footnotes

1 The link to Boas shows that anthropology has different national histories. This is particularly the case regarding histories of subdisciplinary relations. In the U.S. context, the Boasian tradition established an idea of a four-field discipline that, despite Boas's own skepticism about the framework's ability to withstand increasing specialization even in his own day, has managed to be more or less sustained in the United States. As Taussig has articulated elsewhere, important traditions for the kind of work done by anthropologists examining the life sciences can also be found in the British context (Taussig 2006). She traces these to Evans-Pritchard's (1937) analysis of rationality in his study of Zande witchcraft, oracles, and magic and in W. H. R. Rivers's (1922) articulation of the importance of the social in his understanding of rapid population decline in Melanesia. For the purposes of this collection, we point to these traditions in asking what it means to take biology seriously, both as a set of phenomena in nature and as a social practice constituted as a scientific discipline, in light of new genomic knowledge and technologies. We also ask how can and how should the demands of interdisciplinarity and critical engagement be managed.

2 These meetings, organized by Marcia Inhorn and Emily Wentzell and held at Yale University in New Haven, Connecticut, celebrated the 25th anniversary of the Society for Medical Anthropology. A collection of the keynote addresses has recently been published (Inhorn and Wentzell 2012). We are grateful to all who participated in our session on public health genomics.

3 Both Duster (2007) and J. Kahn (2003, 2005) speak to the deeply flawed understandings of race and biology that underlie these arguments.

4 Gilberto Freyre was an influential Brazilian sociologist who, in the 1930s, introduced the idea of Brazil as a “racial democracy” with, he argued, a “mixed race” society being more cohesive, united, and equal.
